# Food allergy clinical course in children and adolescents treated with dupilumab for atopic dermatitis

**DOI:** 10.1016/j.anai.2025.07.023

**Published:** 2025-07-27

**Authors:** Alexander G. Emerson, Matthew S. Krantz, Rachel G. Robison

**Affiliations:** * Division of Pediatric Allergy, Immunology, & Pulmonary Medicine, Department of Pediatrics, Monroe Carell Jr. Children’s Hospital at Vanderbilt, Vanderbilt University Medical Center, Nashville, Tennessee; † Division of Allergy, Pulmonary and Critical Care Medicine, Department of Medicine, Vanderbilt University Medical Center, Nashville, Tennessee; ‡ Department of Biomedical Informatics, Vanderbilt University Medical Center, Nashville, Tennessee

## Abstract

**Background:**

Dupilumab is a monoclonal antibody that inhibits type 2 inflammation through a blockade of interleukin-4 receptor alpha. It is approved for a number of atopic conditions; however, the effect of dupilumab on food allergy has not been fully evaluated.

**Objective:**

To describe the effect of dupilumab use on the clinical course of food allergy in patients receiving dupilumab for comorbid atopic dermatitis.

**Methods:**

This retrospective study reviewed electronic medical records of children with food allergy at a single, tertiary care center receiving dupilumab for the treatment of atopic dermatitis. Food allergy testing, including skin prick test (SPT) and specific IgE (sIgE), and the outcomes of oral food challenges undertaken during routine clinical care were reviewed.

**Results:**

A total of 60 children from 6 months to 18 years of age were included. Linear regression for percent change in measurement by months on dupilumab revealed that sIgE decreased by 0.6% (95% CI −0.8% to −0.4%, *P* < .001) for each additional month of treatment whereas SPT did not differ (0.1% [95% CI −0.6% to 0.7%], *P* = .8). Oral food challenge outcomes in this cohort were similar to previously published cohorts (76% pass rate) undergoing food challenges in a real-world setting irrespective of dupilumab use.

**Conclusion:**

Patients with food allergy receiving dupilumab have greater decreases in sIgE with longer duration of dupilumab treatment whereas SPT seems to be more stable. This suggests that substantial decreases in SPT size over time may be a better correlate of clinical changes in food allergy compared with sIgE in patients with food allergy receiving dupilumab.

## Introduction

Dupilumab is a monoclonal antibody targeting the interleukin-4 receptor alpha (IL-4Rα) chain of the IL-4R and IL-13R. This consequently decreases T_H_2 inflammation by blocking the effects of IL-4 and IL-13 cytokines. Dupilumab is currently approved for the treatment of a number of atopic diseases including asthma, atopic dermatitis (AD), chronic rhinosinusitis with nasal polyposis (CRSwNP), and eosinophilic esophagitis.

One major atopic disorder with an increasing clinical burden for which dupilumab does not have an indication remains food allergy (FA). The National Center for Health Statistics reported in 2013 that the prevalence of FA among children aged 0 to 17 years increased from 3.4% in the late 1990s to 5.1% in the early 2010s.^1^ In 2023, updated data from National Center for Health Statistics estimated the prevalence of FA to be at 5.3% among children aged 0 to 17 years, noting an increasing prevalence with age.^2^ There are scant data in the literature in regard to dupilumab and its effect on FA. It is thought that dupilumab decreases the specific IgE (sIgE) of food allergens by inhibiting IL-4 and IL-13 binding to their receptors, thereby inhibiting class switching to IgE antibodies by B cells and diminishing the proliferation of T_H_2 cells. A cohort study by Spekhorst et al^3^ revealed a sustained decrease in sIgE to food allergens in adult patients while receiving dupilumab for AD. Whether this decrease in sIgE to foods correlates to clinical resolution of FA is yet to be determined.

Dupilumab has been recently investigated as both a monotherapy and as an adjunct with oral immunotherapy (OIT) for treatment of peanut allergy in children. A phase II multicenter, single-arm study of dupilumab as monotherapy on peanut allergy desensitization found that only 8% of subjects could pass a double-blind, placebo-controlled food challenge to the primary end point of 444 mg after 24 weeks of therapy with dupilumab every 2 weeks.^4^ The use of dupilumab as an adjunct to peanut OIT was found to provide a modest increase in OIT efficacy as compared with placebo plus OIT; however, it did not appear to provide protection against OIT-related anaphylaxis.^5^ The effect of dupilumab on food allergens other than peanut is limited to case reports including that of a 30 year old with severe AD and concomitant corn and pistachio allergy prescribed dupilumab for AD who subsequently tolerated corn and pistachio during an open food challenge after 3 months of therapy.^6^ Another case report highlighted a 29-year-old man with recalcitrant AD ultimately started on dupilumab weekly with improvement of his AD. He had a history of severe reactions to several foods with subsequent positive skin prick test (SPT) result and elevated sIgE level to peanut, tree nut, shellfish, and fin fish. His reactions to foods decreased after starting dupilumab, and he was able to pass oral challenges to shellfish, fin fish, and some tree nuts and maintain these foods in his diet.^7^

We hypothesized that dupilumab would have a similar effect on FA in pediatric patients, with a decrease in sIgE and SPT to food allergens, potentially leading to tolerance of previously avoided foods. Therefore, we conducted a retrospective chart review of pediatric patients seen in a single, tertiary care center’s allergy and immunology clinic receiving dupilumab for AD with concomitant FA.

## Methods

### Subject Database

After institutional review board approval, a retrospective review of the electronic medical records of children aged 6 months to 18 years with FA and concomitant AD who were started on dupilumab for treatment of their AD was performed. All patients were seen in the pediatric allergy and immunology clinic at Vanderbilt University Medical Center between June 2019 and July 2024. FA testing, including SPT (Stallergenes Greer, Lenoir, North Carolina) and sIgE in kilounit per liter (ImmunoCAP, Thermo Fisher, Uppsala, Sweden) performed during routine clinical care, was reviewed. The outcome of oral food challenges, both performed in clinic and at home through home introduction or accidental ingestion, was also collected.

**Figure 1.**
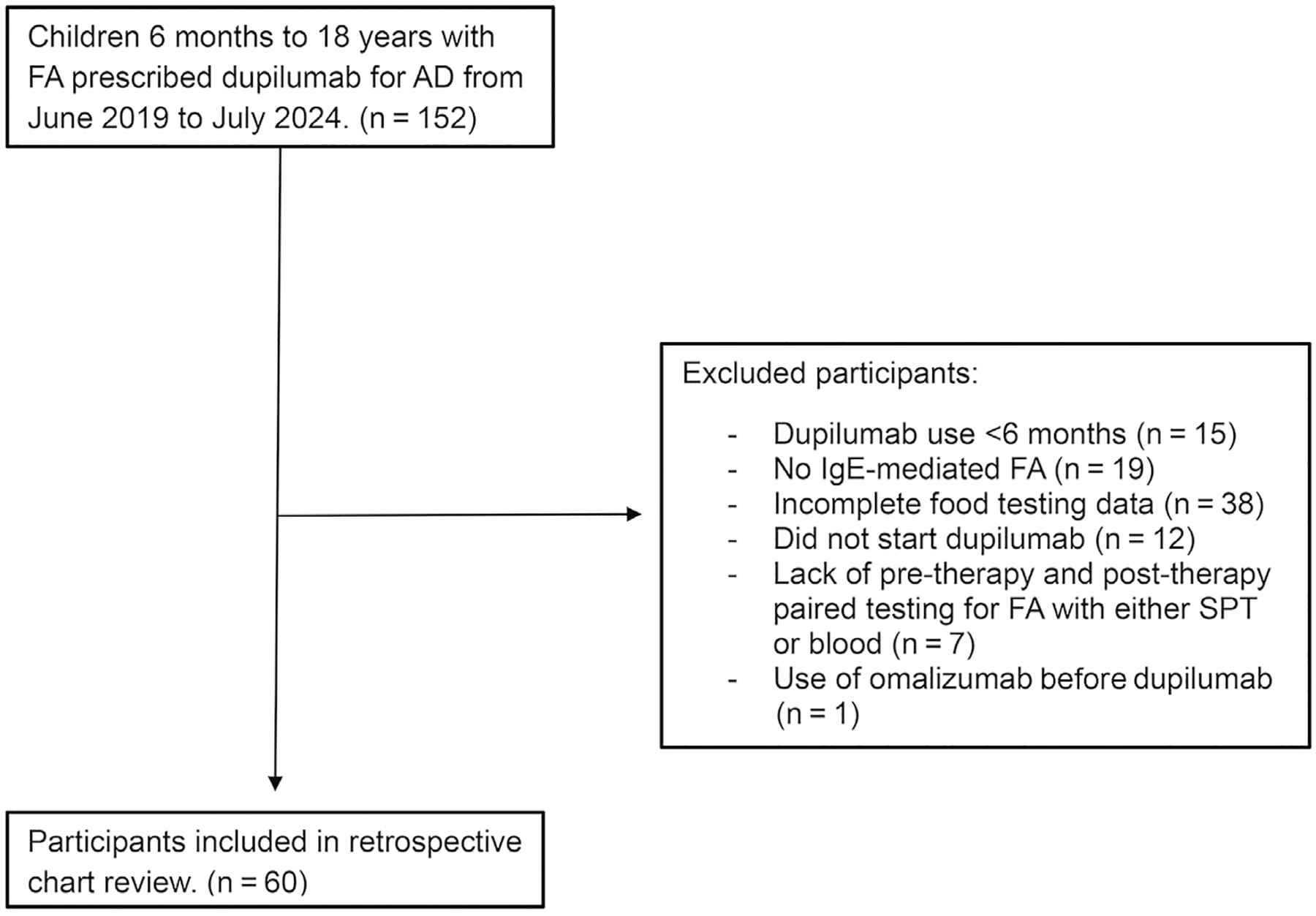
Study participant flow diagram. AD, atopic dermatitis; FA, food allergy; SPT, skin prick testing.

A total of 152 patients were initially identified within the electronic medical records with International Classification of Diseases, 10th Revision, diagnosis codes for eczema and various food allergens (eTable 1) and dupilumab listed as a prescribed medication. Exclusion criteria included the following: dupilumab use less than 6 months, non–IgE-mediated FA, incomplete FA testing (no measurements available from both before and after initiation of dupilumab), and not starting dupilumab despite prescription being ordered by primary allergist. A total of 60 patients were included in the final review (Fig 1).

### Statistical Methods

For foods with which data were collected for at least 10 subjects with either paired SPT and/or sIgE measurements before and after initiation of dupilumab, we determined the mean and 95% CI for percent change and performed a 2-tailed *P* less than .05 *t* test for each food and overall by measurement. We performed linear regression using robust SEs to determine the percent change in measurement by time on dupilumab in months and reported regression coefficients and 95% CI for the SPT and sIgE models. Analyses were performed using R, version 4.4.1 (R Foundation for Statistical Computing).^8^

## Results

A total of 60 subjects met the inclusion criteria for analysis. Subject baseline demographic data, including allergic comorbidities and duration of dupilumab use at time of SPT and sIgE collection, are outlined in Table 1. Most patients were male and had comorbid asthma and allergic rhinitis. All subjects reported a subjective response to dupilumab. Furthermore, 53 subjects (88%) reported positive subjective response, whereas 7 subjects (12%) reported partial subjective response to dupilumab.

**Figure 2.**
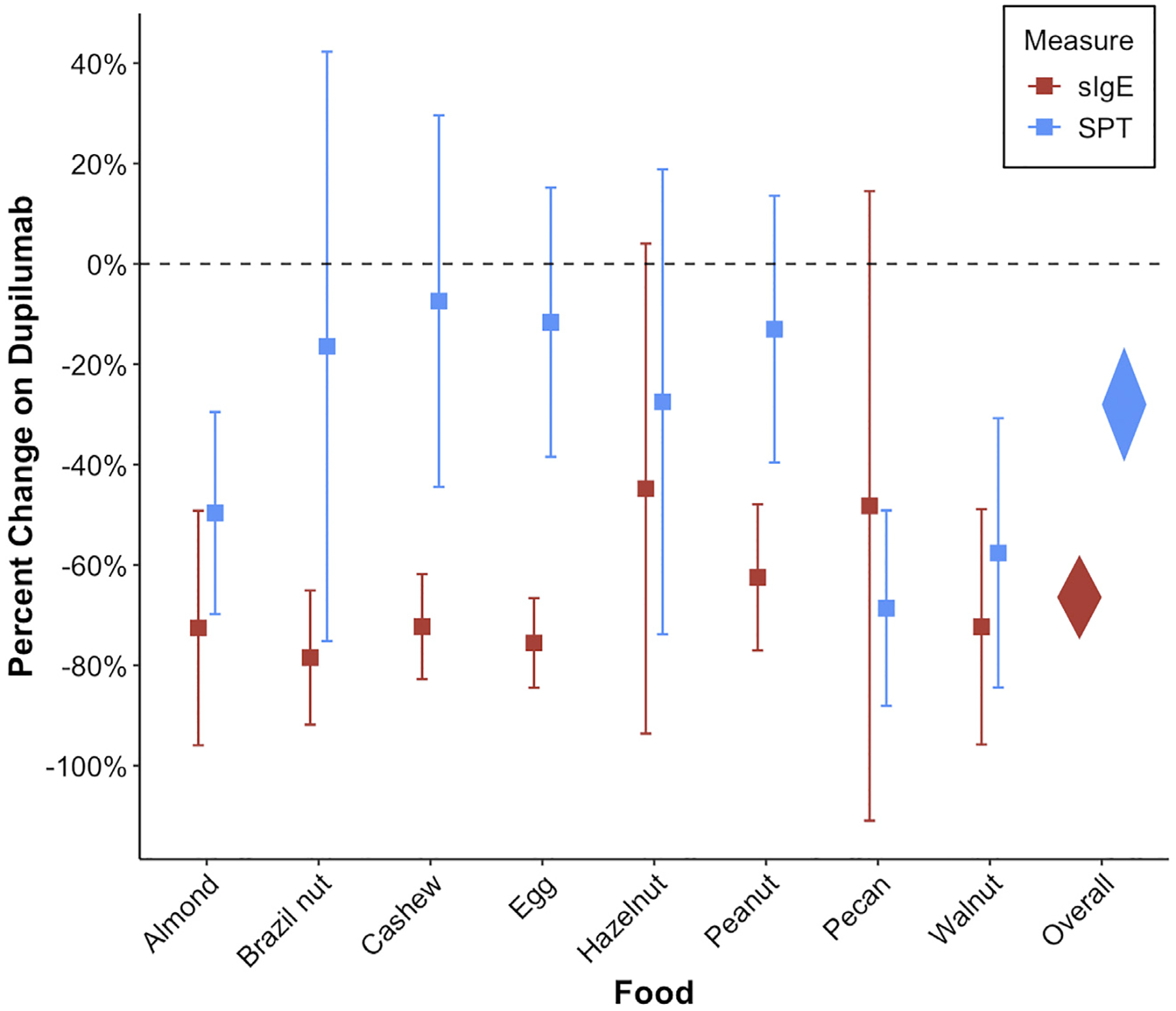
Percent change in SPT wheal size and sIgE while receiving dupilumab by food allergen. sIgE, specific IgE; SPT, skin prick testing.

**Figure 3.**
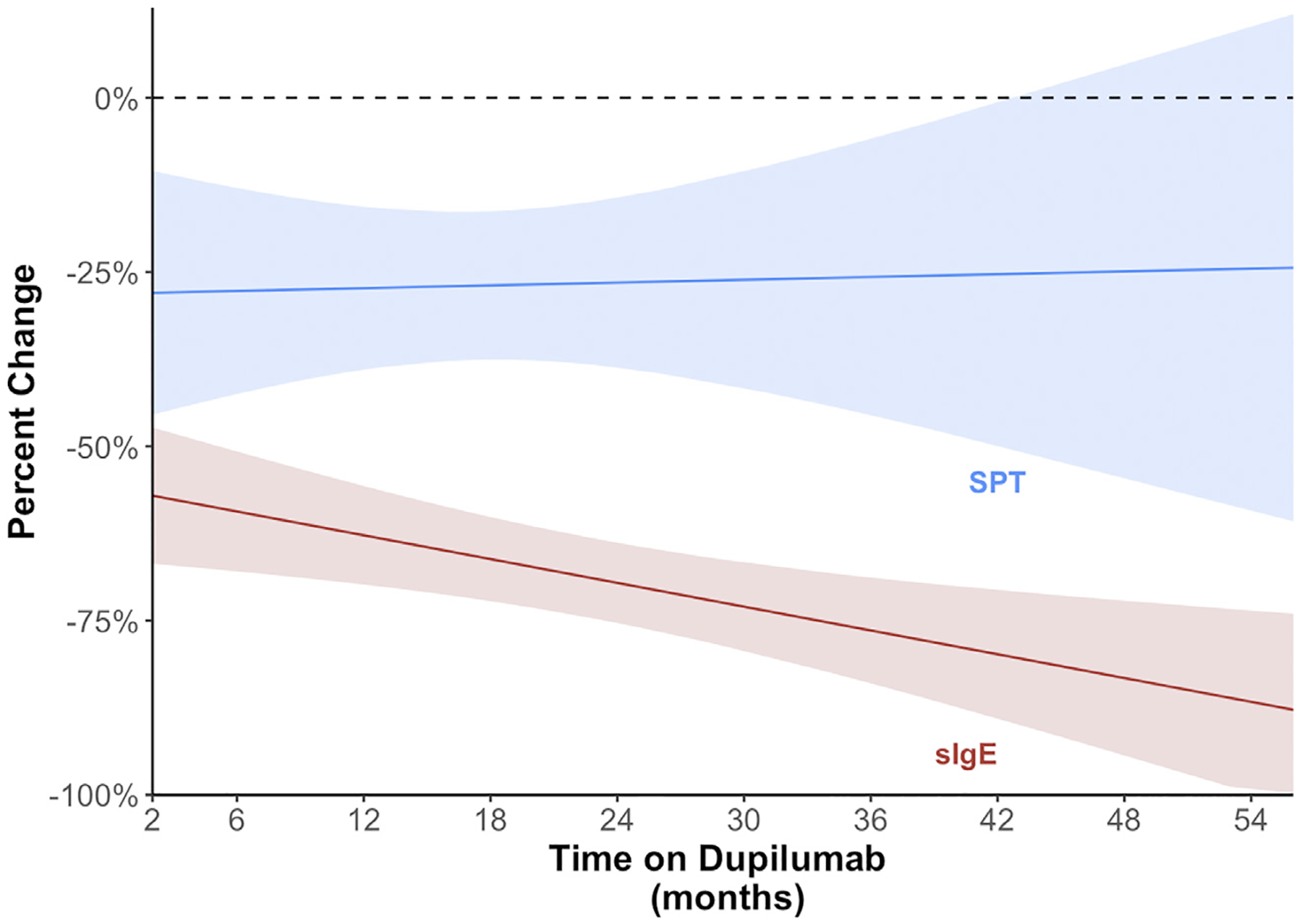
Percent change in overall SPT wheal size and sIgE over time on dupilumab. sIgE, specific IgE; SPT, skin prick testing.

The mean percent decrease overall in patients receiving dupilumab was greater for sIgE (−66.4% [95% CI −74.9%, −57.9%]) compared with SPT (−28% [95% CI −39.4%, −16.5%]) (*P* < .001). In addition, 6 foods (almond, Brazil nut, cashew, egg, peanut, and walnut) demonstrated statistically significant percent decreases in sIgE but only 3 foods (almond, pecan, and walnut) for SPT wheal size in patients receiving dupilumab (Fig 2 and eTable 2). In the linear regression model with robust SEs for percent change in sIgE by months on dupilumab, we found an estimated 0.6% decline in sIgE for every month of additional dupilumab treatment (95% CI −0.8%, −0.4%, *P* < .001) (Fig 3). However, increased time on dupilumab was not associated with statistically significant change in SPT wheal size (0.1% [95% CI −0.6%, 0.7%], *P* = .8).

**Figure 4.**
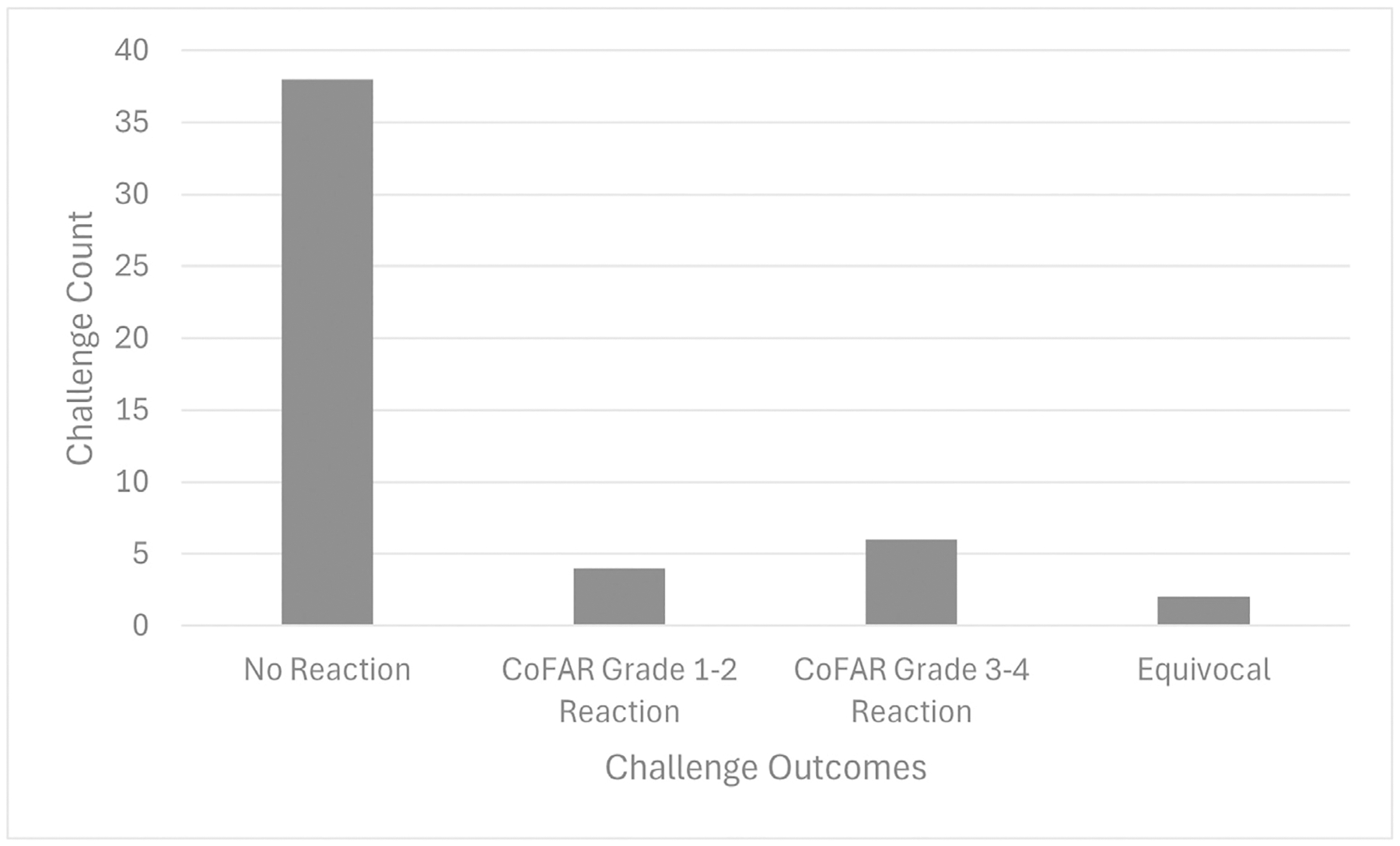
Completed oral food challenge outcomes. No reaction foods challenged: almond (3), beef (1), cashew (2), cow’s milk (1), baked milk (3), crab (1), egg (9), baked egg (4), peanut (5), pecan (2), soy (3), tilapia (1), walnut (1), and wheat (2). CoFAR grade 1–2 reaction foods challenged: egg (1), baked egg (1), and peanut (2). CoFAR grade 3–4 reaction foods challenged: egg (2), baked egg (2), baked milk (1), and peanut (1). Equivocal reaction foods challenged: baked egg (1) and baked milk (1). CoFAR, Consortium for Food Allergy Research.

A total of 50 food challenges occurred among 31 participants. Of these challenges, 38 (76%) resulted in no reaction (ie, pass), 4 (8%) resulted in Consortium for Food Allergy Research grade 1 to 2 allergic reactions treated with antihistamines only, and 6 (12%) resulted in anaphylaxis (Consortium for Food Allergy Research grades 3 to 4 severity) treated with epinephrine (Fig 4).^9^ Furthermore, 2 challenges (4%) (a single accidental home exposure to both baked egg and milk in a cookie) were deemed equivocal resulting in oral itching only; however, the subject continued to avoid both egg and milk due to personal preference. Of the subjects who passed their challenge, 13 were receiving dupilumab every 4 weeks and 11 were receiving dupilumab every 2 weeks. Details of the positive challenges, including occurrences by food allergen, are detailed in Table 2. Of the 7 failed in-office challenges, 4 participants received epinephrine. All 4 of these subjects ingested more than half of the challenge goal before a reaction was found. Furthermore, 3 of these were challenges to egg (2 of which were baked egg). The fourth challenge treated with epinephrine was a subject who consumed 100% of a muffin containing baked milk before having severe anaphylaxis requiring 3 doses of epinephrine.

## Discussion

Dupilumab is a highly effective biologic medication used for the treatment of allergic-mediated inflammation in children with AD and asthma. The effect of dupilumab over time on IgE-mediated FA is only beginning to be studied. Our study revealed that dupilumab induced a significant reduction in food sIgE in pediatric patients with AD and concomitant FA in a median of 17 months. This is in keeping with previous studies in adult patients with AD and FA and in patients with aeroallergen sensitization.^3,10^ Only 2 foods (hazelnut and pecan) did not demonstrate a statistically significant reduction in sIgE with a 95% CI crossing slightly more than 0. We attribute this finding to skewing by outliers in which sIgE level increased to these nuts after the start of dupilumab.

A novel observation found in our study is that dupilumab seems to have a greater percent decrease on sIgE than SPT wheal size and that sIgE continues to decrease with additional time on dupilumab but SPT wheal size does not. A similar observation was also noted by Huber et al^10^ in regard to aeroallergen testing in a cohort of subjects receiving dupilumab for CRSwNP, in which skin prick wheal size was preserved whereas sIgE values declined significantly after initiation of dupilumab. The authors speculated that SPT may be superior in detecting sensitization during dupilumab treatment.^10^ Validation is warranted to replicate this finding as is study to elucidate mechanisms. The frequency of dupilumab dosing and the total dose (including loading dose) may also have an effect on this observed sIgE decline. Further research is warranted to determine any correlation between dupilumab dose and its effect on sIgE decline.

As dupilumab had a larger effect on food sIgE as compared with SPT wheal size over time, we hypothesize that SPT may be a more accurate biomarker to follow FA course in patients receiving dupilumab therapy. Further study is warranted to determine the predictive value of these biomarkers in predicting challenge outcomes during dupilumab therapy. Unfortunately, our cohort did not have enough patients who had undergone food challenge with paired SPT or sIgE testing both before dupilumab start and post-therapy before challenge to make predictions or associations of passed vs failed challenges in these subjects receiving dupilumab.

In our cohort, 76% of the challenges (38/50) were passed without a reaction, 10% of the challenges (5/50) resulted in mild allergic reactions, and 12% of the challenges (6/50) resulted in anaphylaxis requiring epinephrine. The overall outcome profile of these challenges aligns with previously published oral food challenge outcomes in a real-world setting. Mustafa et al^11^ found that 76% of oral challenges were tolerated (passed) and 20.5% experienced a reaction in a retrospective single-center study of pediatric and adult patients who were not necessarily receiving biologic therapy. As similar challenge reaction rates were noted in our cohort, this may suggest that dupilumab use did not necessarily provide protection against reaction. Therefore, the question still remains if the decrease found in food sIgE with dupilumab therapy is truly associated with desensitization. Further prospective study is warranted in larger cohorts to determine the predictive value of these biomarkers in challenge outcomes in patients receiving dupilumab therapy. Additional limitations of this study include its retrospective nature, lack of inclusion of a control arm who did not receive dupilumab, lack of routine clinical use of objective scoring measures for AD, and incomplete food testing data (ie, paired data before dupilumab start and post-initiation of therapy for both SPT and sIgE testing) for some participants.

Dupilumab has proven to be an effective and well-tolerated therapy for children with AD. As more children are started on dupilumab at an early age and given the high comorbidity of FA in children with AD, the interaction of this medication upon the clinical course of FA and upon our ability to test for sensitization to food allergens will need to be considered during clinical management. In this retrospective review of children receiving dupilumab for AD with comorbid FA, we found that SPT wheal size to food allergens is more preserved over time on dupilumab therapy whereas sIgE values decline with continued dupilumab use. Interestingly, this finding has also been observed in aeroallergen testing with dupilumab use in CRSwNP and suggests that SPT may potentially be a more reliable marker of ongoing sensitization to follow in patients’ FA clinical course. Further prospective investigation is warranted to replicate this finding in other cohorts of patients with FA receiving dupilumab.

## Supplementary Material

1

Supplementary data related to this article can be found at https://doi.org/10.1016/j.anai.2025.07.023.

## Figures and Tables

**Table 1:** Demographics

Baseline demographics	Total (n = 60)
Age, y, median (range)	7 (0.5–18)
Male	42 (70)
Non-Hispanic	47 (84)
White	37 (62)
Black/African American	19 (32)
Asian	2 (3)
Multiracial	2 (3)
Allergic comorbidities	
Atopic dermatitis	60 (100)
Asthma	34 (57)
Allergic rhinitis	53 (88)
Eosinophilic esophagitis	8 (13)
Dupilumab duration	
Dupilumab duration of use at time of SPT, mo, median (range)	14.5 (1–57)
Dupilumab duration of use at time of sIgE collection, mo, median (range)	17 (2–60)

Abbreviations: sIgE, specific IgE; SPT, skin prick test.

NOTE. Data are presented as number (percentage), unless otherwise specified.

**Table 2:** Food-Specific Percent Change in SPT and sIgE for Participants Who Failed Oral Challenge

Participant	Food allergen	Time on dupilumab (mo) at challenge	Age (y) at dupilumab start	Pre-dupilumab SPT (mm)	Post-dupilumab SPT (mm)	Percent change in SPT after dupilumab	Pre-dupilumab sIgE (kU/L)	Post-dupilumab sIgE (kU/L)	Percent change in sIgE after dupilumab	Dupilumab dose at challenge	Symptoms experienced during challenge
CoFAR grades 1–2 reaction											
24	Peanut	23	5	14	4	−71%	>100	18.7	−81%	300 mg every 4 wk	Oral pruritus
68	Peanut	19	3	20	8	−60%	2.48	0.36	−85%	200 mg every 4 wk	Urticaria, redness around mouth, sneezing, pruritus of nose, eyes, and ears
81	Baked egg	48	11	10	7	−30%	5.13	0.72	−86%	200 mg every 2 wk	Eyelid angioedema, eczema flare, abdominal pain
94	Egg	16	12	7	5	−29%	5.87	4.31	−27%	200 mg every 2 wk	Delayed facial urticaria (1 h after leaving challenge)
CoFAR grades 3–4 reaction											
19	Egg	13	2	7	5	−29%	10.2	0.76	−93%	300 mg every 4 wk	Sensation of throat swelling, nausea, urticaria, flushing, wheezing
31	Peanut	24	3	N/C	N/C	N/C	91.3	1.98	−98%	200 mg every 2 wk	Rhinorrhea, throat pruritus, urticaria
62	Baked egg	21	5	30	16	−47%	34.7	4.88	−86%	300 mg every 4 wk	Cough, shortness of breath, urticaria, facial angioedema, rhinorrhea
63	Baked egg	21	5	15	15	0%	99.9	10.2	−90%	300 mg every 4 wk	Urticaria, abdominal pain, periorbital angioedema
68	Baked milk	23	3	25	8	−68%	60.2	6.12	−90%	200 mg every 4 wk	Hives, demeanor change, cough, facial/periorbital angioedema, lethargy
79	Egg	8	3	20	11	−45%	42	54.3	+29%	200 mg every 4 wk	Delayed urticaria, angioedema of lips (2–3 h after ingestion)

Abbreviations: CoFAR, Consortium for Food Allergy Research; N/C, not collected; sIgE, specific IgE; SPT, skin prick test.

## References

[1] Centers for Disease Control and Prevention. Trends in allergic conditions among children: United States, 1997–2011. Accessed March 23, 2024. Available at: http://www.cdc.gov/nchs/data/databriefs/db121.pdf.

[2] Zablotsky B, Black LI, Akinbami LJ. Diagnosed allergic conditions in children aged 0–17 years: United States, 2021. NCHS Data Brief. 2023:(459):1–8. 36700870

[3] Spekhorst LS, van der Rijst LP, de Graaf M, van Megen M, Zuithoff NPA, Knulst AC, Dupilumab has a profound effect on specific-IgE levels of several food allergens in atopic dermatitis patients. Allergy. 2023;78(3):875–878. 36420598 10.1111/all.15591

[4] Sindher SB, Nadeau KC, Chinthrajah RS, Leflein JG, Bégin P, Ohayon JA, Efficacy and safety of dupilumab in children with peanut allergy: a multicenter, open-label, Phase II study. Allergy. 2025;80(1):227–237. 39673452 10.1111/all.16404 PMC11724241

[5] Chinthrajah RS, Sindher SB, Nadeau KC, Leflein JG, Spergel JM, Petroni DH, Dupilumab as an adjunct to oral immunotherapy in pediatric patients with peanut allergy. Allergy. 2025;80(3):827–842. 39673367 10.1111/all.16420 PMC11891407

[6] Rial MJ, Barroso B, Sastre J. Dupilumab for treatment of food allergy. J Allergy Clin Immunol Pract. 2019;7(2):673–674. 30075339 10.1016/j.jaip.2018.07.027

[7] Young F, Bhutani T, Otani I, Pham M. Dupilumab as adjunct treatment for persistent food allergy in adulthood. Clin Immunol. 2023;250(suppl):109461.

[8] R Core Team. R: a language and environment for statistical computing. R Foundation for Statistical Computing. Accessed March 23, 2024. Available at: https://www.R-project.org.

[9] Chinthrajah RS, Jones SM, Kim EH, Sicherer SH, Shreffler W, Lanser BJ, Updating the CoFAR grading scale for systemic allergic reactions in food allergy. J Allergy Clin Immunol. 2022;149(6):2166–2170.e1. 35026206 10.1016/j.jaci.2021.12.789 PMC9177543

[10] Huber P, Gröger M, Stihl C, Frankenberger H, Bertlich M, Haubner F, Diagnostics of allergic rhinitis under dupilumab therapy. Eur Arch Oto-Rhino-Laryngol. 2024;281(8):4183–4190. 10.1007/s00405-024-08700-2 PMC11266438 38722319

[11] Mustafa SS, Bress J, Capucilli P, Tuong LA, Denise-Sanchez-Tejera Patrawala S, Outcomes of oral food challenges in a real-world setting, with predictors of outcomes. Ann Allergy Asthma Immunol. 2023;131(5):655–660. 37453573 10.1016/j.anai.2023.07.005

