# Supplementary material for: Food allergy clinical course in children and adolescents treated with dupilumab for atopic dermatitis
Source: Ann Allergy Asthma Immunol. Author manuscript; Available in PMC 2025 Nov 12. (PMC12609014; doi:10.1016/j.anai.2025.07.023)
Supplement: 1 [file NIHMS2121842-supplement-1.pdf]

Supplementary Data

eTable 1
Inclusion Diagnoses and Corresponding ICD-10 Codes

Diagnosis	ICD-10 code
Flexural eczema	L20.82
Infantile (acute) (chronic) eczema	L20.83
Intrinsic (allergic) eczema	L20.84
Other atopic dermatitis	L20.89
Atopic dermatitis, unspecified	L20.9
Atopic dermatitis	L20.*
Dermatitis due to ingested food	L27.2
Allergy to peanuts	Z91.010
Allergy to milk products	Z91.011
Allergy to eggs	Z91.012
Allergy to seafood	Z91.013
Allergy to mammalian meats	Z91.014
Allergy to other foods	Z91.018
Food additives allergy status	Z91.02
Anaphylactic reaction due to unspecified food, initial encounter	T78.00XA
Anaphylactic reaction due to unspecified food, sequela	T78.00XS
Anaphylactic reaction due to unspecified food, subsequent encounter	T78.00XD
Anaphylactic reaction due to peanuts, initial encounter	T78.01XA
Anaphylactic reaction due to peanuts, sequela	T78.01XS
Anaphylactic reaction due to peanuts, subsequent encounter	T78.01XD
Anaphylactic reaction due to shellfish (crustaceans), initial encounter	T78.02XA
Anaphylactic reaction due to shellfish (crustaceans), sequela	T78.02XS
Anaphylactic reaction due to shellfish (crustaceans), subsequent encounter	T78.02XD
Anaphylactic reaction due to other fish, initial encounter	T78.03XA
Anaphylactic reaction due to other fish, sequela	T78.03XS
Anaphylactic reaction due to other fish, subsequent encounter	T78.03XD
Anaphylactic reaction due to fruits and vegetables, initial encounter	T78.04XA
Anaphylactic reaction due to fruits and vegetables, sequela	T78.04XS
Anaphylactic reaction due to fruits and vegetables, subsequent encounter	T78.04XD
Anaphylactic reaction due to tree nuts and seeds, initial encounter	T78.05XA
Anaphylactic reaction due to tree nuts and seeds, sequela	T78.05XS
Anaphylactic reaction due to tree nuts and seeds, subsequent encounter	T78.05XD
Anaphylactic reaction due to food additives, initial encounter	T78.06XA
Anaphylactic reaction due to food additives, sequelae	T78.06XS
Anaphylactic reaction due to food additives, subsequent encounter	T78.06XD
Anaphylactic reaction due to milk and dairy products, initial encounter	T78.07XA
Anaphylactic reaction due to milk and dairy products, sequela	T78.07XS
Anaphylactic reaction due to milk and dairy products, subsequent encounter	T78.07XD
Anaphylactic reaction due to eggs, initial encounter	T78.08XA
Anaphylactic reaction due to eggs, sequela	T78.08XS
Anaphylactic reaction due to eggs, subsequent encounter	T78.08XD
Anaphylactic reaction due to other food products, initial encounter	T78.09XA
Anaphylactic reaction due to other food products, sequela	T78.09XS
Anaphylactic reaction due to other food products, subsequent encounter	T78.09XD
Other adverse food reactions, not elsewhere classified, initial encounter	T78.1XXA
Other adverse food reactions, not elsewhere classified, sequela	T78.1XXS
Other adverse food reactions, not elsewhere classified, subsequent encounter	T78.1XXD

Abbreviation: ICD-10, International Classification of Diseases, 10th Revision.

eTable 2
Mean Food-Specific SPT Wheal and sIgE Percent Change After Dupilumab Initiation

Food allergen	Mean % change in sIgE (95% CI)	Mean % change in SPT (95% CI)	*P* value
Almond	−73% (−96%, −49%)	−50% (−70%, −30%)	.126
Brazil nut	−78% (−92%, −65%)	−16% (−75%, 42%)	.043
Cashew	−72% (−83%, −62%)	−7% (−44%, 30%)	.002
Egg	−76% (−84%, −67%)	−12% (−38%, 15%)	<.001
Hazelnut	−45% (−94%, 4%)	−27% (−74%, 19%)	.592
Peanut	−62% (−77%, −48%)	−13% (−40%, 14%)	.002
Pecan	−48% (−111%, 15%)	−69% (−88%, −49%)	.513
Walnut	−72% (−96%, −49%)	−58% (−84%, −31%)	.385

Abbreviations: sIgE, specific IgE; SPT, skin prick test.
